# Evidence of intraspecific prey switching: stage-structured predation of polar bears on ringed seals

**DOI:** 10.1007/s00442-018-4297-x

**Published:** 2018-11-19

**Authors:** Jody R. Reimer, Hannah Brown, Elaine Beltaos-Kerr, Gerda de Vries

**Affiliations:** 1 Department of Biological Sciences, University of Alberta, Edmonton, Canada; 2 Department of Mathematical and Statistical Sciences, University of Alberta, Edmonton, Canada; 3 Department of Mathematics and Statistics, MacEwan University, Edmonton, Canada

**Keywords:** Age-dependent predation, Marine mammals, Matrix model, Prey switching, Stage-dependent predation

## Abstract

Prey switching is a phenomenon in which a predator disproportionately consumes the most abundant prey type, and switches to preferentially consume another prey type if the first becomes relatively rare. This concept may be expanded outside of its usual usage describing switching between prey species (interspecific), to describe switching between prey stages within a given species (intraspecific). Polar bears (*Ursus maritimus*) are thought to seek out naive ringed seal (*Pusa hispida*) pups in the spring, but how that may change in years with low seal productivity is unknown. We addressed two main questions: If polar bears typically select for ringed seals’ pups, how does this change in years with reduced ringed-seal productivity? How does polar bear predation during years with low ringed-seal productivity impact the ringed seal population? We created a matrix population model for ringed seals to get an estimate of each stage’s availability to polar bears in the spring. These estimates of availability were combined with existing studies on the ages of seals consumed by polar bears in years of both high and low ringed seal productivity. Our results suggest that polar bears typically strongly select for ringed seal pups, but switch to disproportionately select older ringed seals in years with low pup availability. The effects of this on ringed seal population growth appear negligible. Non-intuitive results on the effect of prey switching on the prey population emphasize the importance of considering environmental sequences rather than individual years.

**Electronic supplementary material:**

The online version of this article (10.1007/s00442-018-4297-x) contains supplementary material, which is available to authorized users.

## Introduction

Prey switching is one hypothesized mechanism for stabilizing prey populations, removing predation pressure on a depleted prey population which may allow for that population’s recovery (Murdoch 1969). The phenomenon may be broadly described as one in which a predator preferentially consumes the most abundant prey species, and switches to preferentially consume another if the first species becomes relatively rare (Murdoch 1969). A variety of mechanisms for prey switching have been proposed: the relative vulnerability of prey may change as their frequency changes, a predator may develop a “search image” of the more abundant prey, searching or hunting strategies between prey species may be mutually exclusive, or prey species may be temporally or spatially segregated (Greenwood 1984; Hughes and Croy 1993; Murdoch 1969; Murdoch et al. 1975; Real 1990; Tinbergen 1960). These mechanisms need not be restricted to *interspecific* effects. With slight modification of the previous definition, we describe *intraspecific* prey switching as a scenario in which a predator preferentially consumes the most abundant stage in a species, but switches to preferentially consume another if that stage becomes relatively rare.

Prey species often experience variable predation during different stages in their lives. For example, wolves (*Canis lupus*) prey primarily on juvenile or very old moose (*Alces alces*); Walleye (*Sander vitreus*) prey primarily on juvenile yellow perch (*Perca flavescens*) (Nielsen 1980); and sparrowhawk (*Accipiter nisus* L.) prey primarily on juvenile redshanks (*Tringa totanus*) (Cresswell 1994). Theoretical work on age-specific predation has revealed its complexity, with models showing the inclusion of age-specific predation to be both stabilizing and destabilizing, depending on model structure and parameter values (Hastings 1983, 1984; McNair 1987; Smith and Mead 1974). Little work has been done, however, to explore the response by the predator if there is a sudden reduction in their preferred prey age or stage class. In this paper, we investigate the possibility of intraspecific switching by polar bears (*Ursus maritimus*) between stage classes of their primary prey species, ringed seals (*Pusa hispida*), in years where environmental conditions resulted in an anomalously low number of ringed seal pups.

Ringed seals are the most abundant Arctic pinniped, and can be found throughout the Arctic (Reeves 1998). Ringed seals are the primary food source of polar bears (Smith 1980; Stirling 2002; Stirling and Archibald 1977), and the population sizes of the two species are closely linked throughout their overlapping ranges (Stirling 2002; Stirling and Øritsland 1995). Ringed seals rely on the sea ice as a substrate for pupping, nursing, molting, and mating (Smith 1987; Smith and Stirling 1975) and each of these processes is thus sensitive to fluctuations in ice conditions (Kelly et al. 2010). A causal relationship has been suggested between anomalously late ice breakup in the spring and reduced ringed-seal productivity (Harwood et al. 2012; Stirling 2002). Hypothesized mechanisms include increased energy required to maintain breathing holes through thicker ice, or more general reductions in marine productivity due to reduced light (Forest et al. 2011). Decadal fluctuations in ice breakup and corresponding reductions in ringed seal productivity have been observed in the eastern Beaufort Sea in the mid-1960s, 1970s, 1980s, and early 2000s. Similar concurrence of late ice breakup and reduced ringed-seal productivity has been suggested in Hudson Bay, Canada (Chambellant 2010).

Polar bears prey heavily on ringed seal pups, so in years with low ringed-seal productivity, bears may be forced to change either the composition of their diet, reduce their energy intake, or both. Data on seals killed by polar bears in the eastern Beaufort Sea during spring provide some insight (Pilfold et al. 2012). In years with typical, high ringed-seal productivity, one study found that approximately 70% of observed kills were pups, while in years with late ice breakup and reduced productivity, only 20% of observed kills were pups (Pilfold et al. 2012). How these predation frequencies compare to the availability of each stage is unknown, which leads to the questions we address here: If polar bears typically select for ringed seals’ pups, how does this change in years with reduced ringed-seal productivity? How does polar bear predation during years with low ringed-seal productivity impact the ringed seal population?

While these questions are simple, their answers rely on unknown information about the stage structure and abundance of the seals available to polar bears. Estimating seal availability in this way required careful use of results from several other studies in a logical, if somewhat technical, series of calculations. To estimate prey availability, we created a structured population model for ringed seals. As much as possible, we parametrized our model with values taken from the eastern Beaufort Sea. Since the early 1970s, ringed seals in Amundsen Gulf and Prince Albert Sound have been monitored through a partnership between scientists and Inuvialuit harvesters, providing an extensive body of literature on seals in this area (Harwood et al. 2000, 2012; Kingsley and Byers 1998; Smith 1987; Stirling et al. 1977). We took estimates of both ringed seal and polar bear abundances over both the Southern and Northern Beaufort management subpopulations, as defined by the International Union for Conservation of Nature, Polar Bear Specialist Group (IUCN Polar Bear Specialist Group 2017a).

Assuming that the ratio of different types of prey in a predator’s diet is a good indicator of the predator’s preference (Murdoch 1969), we compared the composition of ringed seal stages killed by polar bears (Pilfold et al. 2012) to each stage’s availability in years of both high and low ringed-seal productivity.

## Methods

In years with late ice breakup, two shifts in demographic responses occur in the seal population: (1) reduced pup production, and (2) changes in survival probabilities resulting from shifts in predation pressure by polar bears. The reduction in pup production between high- and low-productivity years has been documented (Smith 1987), and several estimates of survival probabilities exist for typical years with high productivity (Table 1A). In low-productivity years, however, changes in predation pressure and implications for annual survival probabilities are unknown. We estimated the age-specific predation pressure and survival probabilities in low-productivity years by combining existing empirical studies with results from matrix model theory. Once survival probabilities incorporating predation pressure were obtained for years of both high and low productivity, we could then explore population level effects of age-specific predation.

**Table 1:** Estimates and sources of parameters used in the age-structured matrix model and calculations of predation pressure

Parameter	Values	Description	Sources and notes
*A. Demographic parameters*			
$\sigma _{\mathrm{P}}^H$	0.65	Annual survival by stage; high-productivity years	(Kelly et al. 2010) $\sigma _j^{H_\mathrm{c}} = \sigma _j^H$, all *j*
$\sigma _{\mathrm{J}}^H$	0.9		
$\sigma _{\mathrm{Y}}^H$	0.9		
$\sigma _{\mathrm{M}}^H$	0.9		
$\sigma _{\mathrm{P}}^L, \sigma _{\mathrm{J}}^L, \sigma _{\mathrm{Y}}^L, \sigma _{\mathrm{M}}^L$	See Table 2	Annual survival by stage; low-productivity years	Calculated, Eq. (8)
$$\sigma _{\mathrm{P}}^{L_\mathrm{c}}, \sigma _{\mathrm{J}}^{L_\mathrm{c}}, \sigma _{\mathrm{Y}}^{L_\mathrm{c}}, \sigma _{\mathrm{M}}^{L_\mathrm{c}}$$			
$m_4^H$	0.098	Mean female offspring by age; high-productivity years	Table 26 (Smith 1987) $m_j^H = 0,\,\, j \le 3$ $m_j^{H_\mathrm{c}} = m_j^H$, all *j*
$m_5^H$	0.144		
$m_6^H$	0.195		
$m_7^H$	0.247		
$m_8^H$	0.302		
$m_9^H$	0.353		
$m_{10}^H$	0.401		
$m_{11+}^H$	0.438		
$m_4^L$	0.044	Mean female offspring by age; low-productivity years	Table 26 (Smith 1987) $m_j^L = 0,\,\, j \le 3$ $m_j^{L_\mathrm{c}} = m_j^L$, all *j*
$m_5^L$	0.065		
$m_6^L$	0.088		
$m_7^L$	0.111		
$m_8^L$	0.136		
$m_9^L$	0.159		
$m_{10}^L$	0.167		
$m_{11+}^L$	0.197		
*B. General parameters*			
$\theta _{\mathrm{RS}}$	2/3	Proportion of biomass polar bears obtain from ringed seals	Pilfold et al. (2012)
${\text{mee}}_{\eta}$	See “Appendix A”	Metabolic energetic equivalent for stage $\eta$-polar bear	Table 2 (Regehr et al. 2015)
$p_{\eta}$	See “Appendix A”	Percentage of stage $\eta$ bears in Beaufort Sea	Table 3 (Stirling and Øritsland 1995)
$B_{\mathrm{BS}}$	3000	Number of bears in Beaufort Sea in the 1980s	(IUCN Polar Bear Specialist Group 2017b, c)
FMR	11,375.8 kcal/day	Field metabolic rate for adult female polar bear	Pagano et al. (2018)
$k_{\mathrm{P}}^H$	84	Number of kills of stage-*j* seals; High-productivity years	
$k_{\mathrm{J}}^H$	19		(Pilfold et al. 2012) 120 total observations
$k_{\mathrm{Y}}^H$	9		
$k_{\mathrm{M}}^H$	8		
$k_{\mathrm{P}}^L$	56	Number of kills of stage-*j* seals; Low-productivity years	(Pilfold et al. 2012) 278 total observations
$k_{\mathrm{J}}^L$	60		
$k_{\mathrm{Y}}^L$	81		
$k_{\mathrm{M}}^L$	81		
$k_{\mathrm{P}}^{H_\mathrm{c}}$	78	Hypothetical number of kills of stage-*j* seals, for comparison; high-productivity years hypothetical 120 observations	Calculated; “Hypothetical kill frequencies”
$k_J^{H_\mathrm{c}}$	20		
$k_{\mathrm{Y}}^{H_\mathrm{c}}$	11		
$k_{\mathrm{M}}^{H_\mathrm{c}}$	11		
$k_{\mathrm{P}}^{L_\mathrm{c}}$	181	Hypothetical number of kills of stage-*j* seals, for comparison; low-productivity years hypothetical 278 observations	Calculated; “Hypothetical kill frequencies”
$k_{\mathrm{J}}^{L_\mathrm{c}}$	45		
$k_{\mathrm{Y}}^{L_\mathrm{c}}$	27		
$k_{\mathrm{M}}^{L_\mathrm{c}}$	25		
${\text{cal}}_{\mathrm{P}}$	82,500 kcal		
${\text{cal}}_{\mathrm{J}}$	150,000 kcal	Calories from stage-*j* seals in the spring	Stirling and Øritsland (1995)
${\text{cal}}_{\mathrm{Y}}$	150,000 kcal		
${\text{cal}}_{\mathrm{M}}$	150,000 kcal		
$S_{\mathrm{BS}}$	500,000	Number of female seals in Beaufort Sea in the 1970–1990s	Stirling and Øritsland (1995)

Demographic parameters classified by stage (pups, *P*; juveniles, *J*; young adults, *Y*; mature adults, *M*) rather than by age are used for each age class within the given stage (e.g. since ages 1 through 6 are all classified as juveniles, $\sigma _1$ through $\sigma _6 = \sigma _{\mathrm{J}}$). The polar bear population is divided into eight distinct polar bear stages, so $\eta =$ cubs of the year, yearlings, 2-year-old males and females, subadult males and females, and adult males and females, as in (Regehr et al. 2015). For additional details, see “Appendix A”

**Fig. 1:**
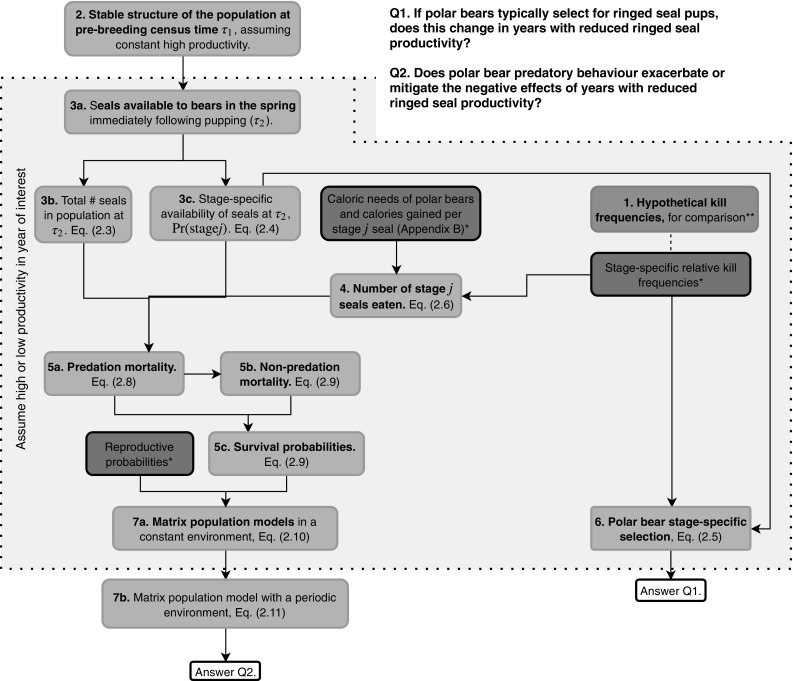
Flow chart of calculations and values required for this study. Quantities drawn from existing literature (values in Table 1A, B) are shown in blue boxes (and denoted with *). The green box (also denoted with **) represents values which have been included as an alternative against which to compare the results obtained using the connecting blue box. All other boxes, in yellow, represent quantities which we calculated in this paper. All boxes included in the shaded area with the dotted border need be calculated for both high- and low-productivity years, both using observed kill frequencies and then using hypothetical constant kill frequencies for comparison. Our methods are organized to correspond to the box numbers, and Methods subsections are ordered accordingly (e.g. box 1 is described in “Hypothetical kill frequencies”, box 2 in “Stable structure of the population at pre-breeding census”, etc.). Census times $\tau _1$ and $\tau _2$ are as in Fig. 2 (color figure online)

The set-up of our study is illustrated in Fig. 1. Methods are described in the order in which they had to be carried out (i.e. working downwards through Fig. 1), so that all of the necessary components required for a given calculation are described prior to them being needed. It may help the reader, however, to know that these methods were designed in the opposite direction, starting with the questions and then filling in any gaps as required (i.e. building upwards in Fig. 1). Prior to this study, a main component needed to answer the two questions of interest was missing, namely the composition of seals available to polar bears in the spring following pupping (box 3c in Fig. 1).

Our second research question tacitly implies a comparison between observed polar bear foraging behaviour and alternate behaviour patterns, against which mitigation or exacerbation may be compared. Our null hypothesis (“Hypothetical kill frequencies”) was that the stage composition of polar bear kills was constant for all years, regardless of fluctuations in ringed-seal productivity. This would imply that in years with low ringed-seal productivity, the fewer pups which were born would experience higher than usual predation and thus lower survival.

To explore the effects of predation, we considered four scenarios (described below). Regardless of the scenario, we first assumed that there had been a series of high-productivity years and estimated the resulting ringed seal population distribution (“Stable structure of the population at pre-breeding census”). We then considered the year following this series of high-productivity years, considering both the case that it was another high-productivity year, but also that it was a year with low productivity. We performed a series of calculations (“Seals available to bears in the spring”, “Number of stage* j* seals eaten”, “Predation mortality, non-predation mortality, and survival”, “Polar bear stage-specific selection”, “Matrix population models”) for the chosen scenario (denoted $\xi$) in a given year. Each scenario encapsulated both ringed seal productivity (either high or low, as determined by that year’s ice conditions), and the composition of polar bear kills (either observed or our proposed comparison hypothesis). Thus much of the work described in “Seals available to bears in the spring”, “Number of stage* j* seals eaten”, “Predation mortality, non-predation mortality, and survival”, “Polar bear stage-specific selection” and “Matrix population models” (the shaded area in Fig. 1) was repeated for each of the four scenarios: high productivity with observed kills, $\xi = H$; low productivity with observed kills, $\xi =L$; and high or low productivity with hypothetical kills for comparison, $\xi = H_\mathrm{c}$ or $L_\mathrm{c}$.

Several assumptions were necessary for the construction of the age-structured population model. We assumed a maximum ringed seal age of 40 years (Lydersen and Gjertz 1987; McLaren 1958) and a 1:1 sex ratio at birth (Lydersen and Gjertz 1987; McLaren 1958). Being a weakly polygynous species Smith and Hammill (1981), we assumed males are sufficiently abundant for reproduction, and so a female-only model is adequate for understanding population dynamics. Annual age-specific reproductive probabilities $m_{i}^\xi$ (see Table 1) were assumed to depend only on the ice conditions of a given year, so $m_{i}^H = m_{i}^{H_\mathrm{c}}$ and $m_{i}^L = m_{i}^{L_\mathrm{c}}$ for $i = 0,\ldots ,40$. The age-specific survival probabilities $\sigma _i^\xi$ of ringed seals in high-productivity years were taken from the literature and thus assumed to be the same regardless of the composition of polar bear kills in those years, i.e. $\sigma _{i}^{H} = \sigma _i^{H_\mathrm{c}}$. The survival probabilities of ringed seals in low-productivity years were not known from the literature, and indeed depended on how polar bears changed their predatory behaviour as reflected in the composition of their kills, so both $\sigma _i^L$ and $\sigma _i^{L_\mathrm{c}}$ (note $\sigma _i^L \ne \sigma _i^{L_\mathrm{c}}$) needed to be derived. We also assumed that ringed seal mortality had two independent sources: predation mortality and non-predation mortality. We assumed predation mortality varied for different environmental states $\xi$, but that non-predation mortality was constant.

**Table 2:** Select stage-specific results

Description	Scenario $\xi$	Value by stage				Equation
		P	J	Y	M	
Hypothetical number of kills of stage *j*	$H_\mathrm{c}$	78	20	12	11	“Hypothetical kill frequencies”
Ringed seals, ${k_j}^\xi$	$L_\mathrm{c}$	181	46	27	25	
${\Pr (\hbox {stage } j)}\;{\mathrm{at}} \,\tau _2$	*H*	0.16	0.45	0.33	0.06	(4)
	*L*	0.08	0.49	0.36	0.07	
Observed proportion of calories	*H*	0.56	0.23	0.11	0.10	(14)
from stage *j*-ringed seals	*L*	0.12	0.24	0.32	0.32	
# stage-*j* seals eaten ($\times 10^3$)	*H*	52.1	11.9	5.6	5.0	(5)
	*L*	11.3	12.1	16.4	16.4	
	*H*	0.29	0.02	0.02	0.07	(7)
Predation mortality	*L*	0.14	0.02	0.04	0.23	
$\Pr (\text{eaten }|\text{stage } j)$	$H_\mathrm{c}$	0.26	0.02	0.02	0.09	
	$L_\mathrm{c}$	0.37	0.02	0.02	0.09	
Non-predation mortality	*H* and *L*	0.08	0.08	0.09	0.03	(8)
	$H_\mathrm{c}$ and $L_\mathrm{c}$	0.12	0.08	0.08	0.01	
Annual survival, low-productivity years, ${\sigma _j}^L, {\sigma _j}^{L_\mathrm{c}}$	*L*	0.79	0.90	0.87	0.75	(8)
	$L_\mathrm{c}$	0.37	0.90	0.90	0.90	

*H* and *L* refer to years of high or low productivity. For comparison, $H_\mathrm{c}$ and $L_\mathrm{c}$ also refer to years with high or low productivity, but with the composition of polar bear kills held constant (see “Hypothetical kill frequencies”). *P*, *J*, *Y*,  and *M* refer to pups, juveniles, young adults, and mature adults respectively. Recall that annual survival probabilities for years with high productivity, $\sigma ^H = \sigma ^{H_\mathrm{c}}$, were taken from existing literature (Table 1)

Note that we required estimates of demographic rates for seals of each age *i*. However, available data on polar bear predation (Pilfold et al. 2012) had a resolution of different life history stages, rather than ages. Where necessary, we thus considered the same four distinct life-history stages *j* as (Pilfold et al. 2012), defined by the ages they encompass: pups (age 0+), juveniles (1–6), young adults (7–20), and mature adults (21–40), denoted as P, J, Y,  and M throughout.

### Hypothetical kill frequencies

We began by constructing a hypothetical distribution of polar bear kills, against which to compare the observed distributions. We chose to compare the observed kill distributions to a scenario in which the distribution of polar bear kills did not depend on ringed seal availability (i.e. whether there are high or low abundances of ringed seal pups in a given year). In this comparison scenario, ringed seal productivity still varied between high and low years, but the composition of polar bear kills was constant. This constant composition of polar bear kills was calculated as the weighted average of the observed kill compositions in years with high and low productivity for a cycle of a given length. Thus the values used in the comparison case depended on the assumed length of environmental cycle, here taken to be 10 years to reflect the decadal environmental cycles observed in the eastern Beaufort Sea.

For example, since ringed seal pups made up 70% of the kills in high-productivity years, and 20% in low-productivity years (Pilfold et al. 2012), the weighted average for a cycle with nine high-productivity years followed by one low-productivity year was $(70 \times 9 + 20)/10 = 65 \%$. To obtain the number of seals we would expect to observe in a sample the same size as in (Pilfold et al. 2012), we multipled by the corresponding sample sizes to get the number of stage-*j* seal kills $k_j^{H_\mathrm{c}}$ and $k_j^{L_\mathrm{c}}$ (Table 1B). Note that the composition of polar bear kills (proportions) will be constant across years, but $k_j^{H_\mathrm{c}} \ne k_j^{L_\mathrm{c}}$ because of the two different sample sizes assumed for consistency with (Pilfold et al. 2012).

### Stable structure of the population at pre-breeding census

**Fig. 2:**
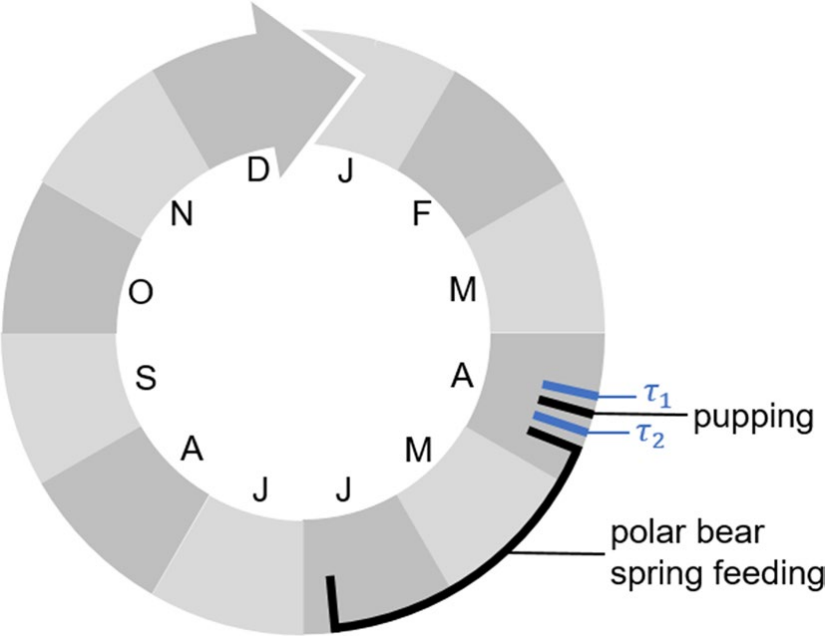
Annual pre- and post-breeding census times $\tau _1$ and $\tau _2$ for the model of ringed seals in the Beaufort Sea, and their relation to key annual events. Census time *t* in Eq. (1) corresponds to $\tau _1$

We began modelling by constructing an age-structured matrix model for ringed seals assumed to experience a constant environment with high productivity. We used an annual pre-breeding census ($\tau _1$ in Fig. 2), immediately before April 15, which has been suggested as the nominal day of peak pupping in the Beaufort Sea (Kingsley and Byers 1998; Smith 1987).

The population size and structure at time *t*, ${\mathbf {x}}(t) = \left[ x_0(t), x_1(t), \ldots , x_{40}(t)\right] ^\intercal$, evolved according to,

1 $$\begin{aligned} {\mathbf {x}}(t+1) = {\mathbf {A}}^H {\mathbf {x}}(t), \end{aligned}$$

where ${\mathbf {A}}^H$ was a Leslie matrix describing the demographic rates for the year preceding time $t+1$, with reproductive rates in the first row, transition probabilities on the subdiagonal, and zeros everywhere else,

2 $$\begin{aligned} {\mathbf {A}}^H=\begin{bmatrix} 0& \cdots& m_4^H \sigma _0^H& \cdots& m_{40}^H \sigma _0^H \\ \sigma _1^H& \ddots& & & 0 \\ 0& \ddots& \ddots & & 0 \\ \vdots& & \ddots& \ddots& \vdots \\ 0& \cdots& \cdots& \sigma _{40}^H& 0 \end{bmatrix}. \end{aligned}$$

Age-specific transition probabilities for an age *i* individual were $\sigma _{i+1}^H$, where $\sigma _{i}^{H}$ was the annual survival probability of an age *i* individual in a high-productivity environment. This subtle indexing point results from the pre-breeding census; following census, individuals first advanced one age class and then survived the year. By this same logic, each age *i* seal gave birth immediately following the census, depending on whether their pregnancy was successful over the past year (i.e. while they were age *i*). The pup then had to survive the year to be counted in the following census. Thus the age-specific annual reproductive rate for an age *i* seal was the product $m_{i}^{H}\, \sigma _0^H$, where $m_{i}^{H}$ is the expected number of offsprings per age *i* seal. Parameter estimates for all entries of ${\mathbf {A}}^H$ were available in the literature (Table 1A).

Assuming a long run of consecutive high-productivity years, we approximated the long-time stable age distribution of the population at $\tau _1$ by calculating the right eigenvector ${\mathbf {w}}$ of ${\mathbf {A}}^H$ corresponding to the dominant eigenvalue, where $\sum _{\nu = 0}^{40}{w_i}=1$. We assumed that the seal population size was constant ($S_{\mathrm{BS}}$), and that it was at this stable age distribution at $\tau _1$ in the year of interest, so ${\mathbf {x}}(\tau _1) = {\mathbf {w}}\, S_{\mathrm{BS}}$.

We explored the appropriateness of assuming the population was at this stable age distribution ${\mathbf {w}}$ by running 10,000 simulations over a range of plausible perturbations from the stable age distribution to explore convergence rates. At the start of each simulation, a random matrix ${\mathbf {A}}_\mathrm{r}$ was generated with nonzero entries randomly selected from [0, 1] in the same locations as in the Leslie matrix, Eq. (2). This encompassed a wide range of plausible fluctuations in demographic rates, but assumed that physiological constraints prevent changes in ringed seal life history. The perturbed age distribution was then $\hat{{\mathbf {w}}}(0) = {\mathbf {A}}_\mathrm{r} \, {\mathbf {w}}/ ||{\mathbf {A}}_\mathrm{r} \, {\mathbf {w}}||_1$ (where $|| \cdot ||_1$ is the L1 norm, the sum of each element in the vector). We then simulated the known convergence of $\hat{{\mathbf {w}}}$ to ${\mathbf {w}}$ following $\hat{{\mathbf {w}}}(t+1) = {\mathbf {A}}^H \hat{{\mathbf {w}}}(t) / ||{\mathbf {A}}^H \hat{{\mathbf {w}}}(t)||_1$, and assessed visually.

### Seals available to bears in the spring

Having approximated the population distribution immediately before pupping in any given year with ${\mathbf {w}}$, we could then calculate the post-pupping seal distribution in a given year with scenario $\xi$. Recall that “Seals available to bears in the spring”, “Number of stage* j* seals eaten”, “Predation mortality, non-predation mortality, and survival”, “Polar bear stage-specific selection” and “Matrix population models” had to be completed for each $\xi$ in $\left\{ H, L, H_\mathrm{c}, L_\mathrm{c}\right\}$. We focused on seal availability immediately following seal-pupping because this is what is available to polar bears in the spring when they consume the majority [up to 80% (Stirling and Øritsland 1995)] of their annual calories. We introduced a second census time $\tau _2$ (Fig. 2) immediately following pupping in the year under consideration. This second census time allowed for the inclusion of density-dependent survival, allowing predation mortality to depend on the size of each stage class. Between $\tau _1$ and $\tau _2$, we assumed that each seal transitions from age *i* to $i+1$ (i.e. grows one year older) and reproduces, but that no mortality occurs. The population at time $\tau _2$ provided an estimate of two desired quantities: total seal abundance, and the population stage structure available to polar bears in the spring.

Our eventual goal was a Leslie matrix ${\mathbf {A}}^\xi$ of the same form as Eq. (2) for each scenario $\xi$. We decomposed $\mathbf{A }^\xi$ into $\mathbf{A }^\xi = \mathbf{A }_2^\xi \mathbf{A }_1^\xi$, so ${\mathbf {x}}(\tau _1+1) = {\mathbf {A}}_2^\xi {\mathbf {A}}_1^\xi {\mathbf {x}}(\tau _1)$. This decomposition of ${\mathbf {A}}^\xi$ into ${\mathbf {A}}_1^\xi$ and ${\mathbf {A}}_2^\xi$ allowed for the entries of ${\mathbf {A}}_2^\xi$ to depend on that year’s productivity, the outcome of ${\mathbf {A}}_1^\xi$ acting on ${\mathbf {x}}$.

$\mathbf{A }_1^\xi$ described the events occurring immediately following $\tau _1$ (i.e. seals reproduce and grow one year older),

$$\begin{aligned} {\mathbf {A}}_1^\xi = \begin{pmatrix} 0 &{} 0 &{} \cdots &{} m_4^\xi &{} \cdots &{} m_{40}^\xi \\ 1 &{} 0 &{} \cdots &{} &{} &{} \\ 0 &{} 1 &{} \cdots &{} &{} &{} \\ \vdots &{} &{} \cdots &{} 0 &{} 1 &{} 0 \end{pmatrix}, \end{aligned}$$

so ${\mathbf {x}}(\tau _2) = {\mathbf {A}}_1^\xi {\mathbf {x}}(\tau _1) \approx {\mathbf {A}}_1^\xi {\mathbf {w}}$. We knew $m_{i}^\xi$ for each $\xi$ from the literature (Table 1A), so each ${\mathbf {A}}_1^\xi$ was known. The abundance and distribution of ringed seals available to bears immediately following pupping (at $\tau _2$ of the given year) was thus

3 $$\begin{aligned} (\text{total} \# \text{ seals } \text{ in } \text{ population })^\xi = ||{\mathbf {x}}(\tau _2)||_1 \end{aligned}$$

and

4 $$\begin{aligned} \Pr \left( \text{stage} {} { j}\right) ^\xi = \sum _\upsilon {x_\upsilon (\tau _2)}/ ||{\mathbf {x}}(\tau _2)||_1, \end{aligned}$$

where $\upsilon$ runs through all ages included in stage-*j* [i.e. pups (age 0+), juveniles (ages 1–6), young adults (ages 7–20), and mature adults (ages 21–40)]. The survival of each stage over the remainder of the year, from $\tau _2$ to $(\tau _1+1)$, was described by ${\mathbf {A}}_2^\xi$,

$$\begin{aligned} {\mathbf {A}}_2^\xi = \begin{pmatrix} \sigma _0^\xi &{} 0 &{} \cdots &{} 0 \\ 0 &{} \sigma _1^\xi &{} \cdots &{} 0 \\ \vdots &{} &{} \ddots &{} \\ 0 &{} \cdots &{} &{} \sigma _{40}^\xi \\ \end{pmatrix}. \end{aligned}$$

### Number of stage-*j* seals eaten

Having calculated the total number of seals in the population at $\tau _2$ and the stage distribution of those seals (Eqs. 3, 4), we still required the total number of seals in each stage consumed by polar bears to eventually calculate predation mortality (Fig. 1). For each stage, *j*, we estimated the number of stage-*j* seals consumed by polar bears by combining relative predation frequencies with studies on the caloric requirements of polar bears and the caloric values of ringed seals. Our estimate (see “Appendix B” for technical derivation details) followed

5 $$\begin{aligned} \# \text{ stage- } {} { j} \text{ seals } \text{eaten}^\xi = \frac{\sum _\eta (365\, p_{\eta} \, B_{\mathrm{BS}}\, \text{ FMR }\, {\text{mee}}_{\eta} )\, k_j^\xi }{\sum _\ell k_\ell ^\xi \, {\text{cal}}_\ell }, \end{aligned}$$

where $\eta$ runs through eight distinct polar bear stages (see Regehr et al. 2015), $\ell$ runs through the four ringed seal stages, and with parameter estimates and descriptions as in Table 1B. Intuitively, this was derived by calculating the total number of calories polar bears in the Beaufort Sea gain from stage-*j* seals annually, and then dividing by the calories gained per individual stage-*j* seal.

### Predation mortality, non-predation mortality and survival

We then had all of the pieces necessary to calculate stage-specific predation mortality $\Pr \left( \text{eaten}\, | \,\text{stage} {} { j}\right) ^\xi$. This is the annual probability that a seal is eaten given it is in stage $j \in \left\{ P, J, Y, M\right\}$. Information available on stage-specific predation, however, was of the form $\Pr \left( \text{stage} { j}\, | \,\text{eaten}\right) ^\xi$ (Pilfold et al. 2012). We use Bayes theorem to obtain the one from the other, expressed as

6 $$\begin{aligned} \Pr \left(\text{eaten}\, | \,\text{stage} {} { j}\right) ^\xi = \frac{\Pr \left(\text{stage} { j}\, | \,\text{eaten}\right) ^\xi \Pr \left(\text{eaten}\right) ^\xi }{\Pr \left(\text{stage} {} { j}\right) ^\xi }. \end{aligned}$$

We substituted

$$\begin{aligned} \Pr \left(\text{stage} {} { j}\, | \,\text{eaten}\right) ^\xi = (\# \text{ stage- } {} { j} \text{ seals } \text{eaten})^\xi /(\text{total} \# \text{ seals } \text{eaten})^\xi \end{aligned}$$

and

$$\begin{aligned} \Pr \left(\text{eaten}\right) ^\xi = (\text{total} \# \text{ seals } \text{eaten})^\xi /(\text{total} \# \text{ seals } \text{ in } \text{ population })^\xi \end{aligned}$$

into Eq. (6). This yielded

7 $$\begin{aligned}&\Pr \left(\text{eaten}\, | \,\text{stage} {} { \, j}\right) ^\xi = \nonumber \\&\quad \frac{\# \text{ stage- } {} { j} \text{ seals } \text{eaten}^\xi }{(\text{total}\, \# \text{ seals } \text{ in } \text{ population })^\xi \Pr \left(\text{stage} {} {\, j}\right) ^\xi }. \end{aligned}$$

We had already calculated the three factors on the right-hand side of Eq. (7) in Eqs.(3) through (5).

Since we assumed that annual survival depends on avoiding two independent sources of mortality, non-predation mortality and mortality due to bear predation,

8 $$\begin{aligned}&\sigma _i^\xi = \nonumber \\&\quad (1 - \text{ non-predation } \text{ mortality }_i^\xi )(1 - \underbrace{{\mathrm{predation mortality}}_i^\xi }_{\Pr \left(\text{eaten}\, | \,\text{stage} {} { j}\right) ^\xi }), \end{aligned}$$

for each age and corresponding stage. Recall that annual survival values ($\sigma _{i}^{H}$ and $\sigma _i^{H_\mathrm{c}}$) for high-productivity years were assumed from the literature (Table 1A), so once we have calculated $\Pr \left(\text{eaten}\, | \,\text{stage} {} { j}\right) ^H$ from Eq. (7), we solved for ($\text{ non-predation } \text{ mortality }^H_i$). Because we assumed that non-predation mortality does not depend on the timing of ice breakup and is approximately constant across years, then $(\text{ non-predation } \text{ mortality }^\xi _i) = (\text{ non-predation } \text{ mortality }^H_i)$ for $\xi = H_\mathrm{c}, L, L_\mathrm{c}$. Using $\Pr \left(\text{eaten}\, | \,\text{stage} { j}\right) ^L$ and $\Pr \left(\text{eaten}\, | \,\text{stage} { j}\right) ^{L_\mathrm{c}}$ as calculated from Eq. (7), we then obtained $\sigma _i^L$ and $\sigma _i^{L_\mathrm{c}}$, which included both the effects of reduced ringed-seal productivity as well as resultant changes in predation mortality.

### Polar bear stage-specific selection

“Hypothetical kill frequencies”, “Stable structure of the population at pre-breeding census” and “Seals available to bears in the spring” included all of the components required to address the first of our two main questions, that of polar bear predation preference in high- versus low-productivity years (Q1 in Fig. 1). We defined selection on each stage *j* for each scenario $\xi$ as

9 $$\begin{aligned} \text{ selection }_j^\xi = \frac{\text{ Proportion } \text{ predated }}{\text{ Proportion } \text{ available }} = \frac{(k_j^\xi /\sum _\ell k_\ell ^\xi )}{\Pr \left( \text{stage} {} { j}\right) ^\xi }, \end{aligned}$$

where $\ell$ runs through the four ringed seal stages, and $\Pr \left( \text{stage} {} { j}\right) ^\xi$ is as in Eq. (4). If $\text{ selection }_j^\xi < 1$, this may be interpreted as polar bears preying on proportionally fewer stage-*j* individuals than what are available. If $\text{ selection }_j^\xi = 1$, this suggests polar bears are preying on stage-*j* seals with the same frequency with which seals in stage *j* occur in the population. If $\text{ selection }_j^\xi > 1$, polar bears are predating more on stage-*j* seals than their relative frequency in the population.

### Matrix population models

Using the results from sections “Hypothetical kill frequencies”, “Stable structure of the population at pre-breeding census”, “Seals available to bears in the spring”, “Number of stage* j* seals eaten” and “Predation mortality, non-predation mortality, and survival”, we addressed our second question (Q2 in Fig. 1). All parameters $\sigma _i^\xi$ and $m_{i}^\xi$ for each scenario $\xi$ had been estimated either from the literature or through our calculations. Thus we formed four Leslie matrices ${\mathbf {A}}^H, {\mathbf {A}}^L, {\mathbf {A}}^{H_\mathrm{c}}$ and ${\mathbf {A}}^{L_\mathrm{c}}$, each of the form (2) but with entries corresponding to $\xi$. Recall that $\sigma _{i}^{H} = \sigma _i^{H_\mathrm{c}}$ and $m_{i}^H = m_{i}^{H_\mathrm{c}}$, so ${\mathbf {A}}^H = {\mathbf {A}}^{H_\mathrm{c}}$.

If we assumed a constant environment $\xi$, the population evolved according to

10 $$\begin{aligned} {\mathbf {x}}(t+1) = {\mathbf {A}}^\xi {\mathbf {x}}(t), \quad \quad \xi = H, \,L, \,H_\mathrm{c}, \,L_\mathrm{c}. \end{aligned}$$

To determine the impact of the decadal cycles suggested to occur in the Beaufort Sea with a periodic matrix model, we assumed a periodic environment over 10 years, characterized by nine years with high productivity, followed by one year with low productivity. One cycle for the scenario with observed polar bear kill proportions was described by ${\mathbf {B}} = {\mathbf {A}}^L\left( {\mathbf {A}}^H\right) ^9$, so

11 $$\begin{aligned} {\mathbf {x}}(t+10) = {\mathbf {B}}\, {\mathbf {x}}(t). \end{aligned}$$

Similarly, for the case with the hypothetical comparison kill proportions, ${\mathbf {B}}_\mathrm{c} = {\mathbf {A}}^{L_\mathrm{c}}\left( {\mathbf {A}}^{H_\mathrm{c}}\right) ^9$.

We calculated the long-term growth rate and age distribution of a population subject to each constant environment, ${\mathbf {A}}^H$, ${\mathbf {A}}^L$, and ${\mathbf {A}}^{L_\mathrm{c}}$, as well as the periodic environments ${\mathbf {B}}$ and ${\mathbf {B}}_\mathrm{c}$ by calculating the matrices’ dominant eigenvalues ($\lambda$) and corresponding right eigenvectors (see Caswell (2001) for a good overview). A negative population growth rate (i.e. $\log \lambda < 0$) implies population decline, and $\log \lambda > 0$ implies long-term exponential population growth. We addressed our second question through the analysis and comparison of these matrix models between the scenario with observed kill frequencies and the scenario with the hypothetical frequencies (Fig. 1).

### Sensitivity to model parameters

This work relied on model parameters taken from the relevant literature, which introduced several sources of uncertainty into the results. To better understand this, we performed both qualitative and quantitative sensitivity analyses where appropriate. To explore the sensitivity of the answer to Question 1 (Fig. 1), on polar bear selection, we varied all parameters which contribute to the answer to Question 1 (all parameters in Table 1A) by ± 5%, observing if the selection pattern qualitatively changed. The other parameters used in this study (Table 1B) all contributed to the answer to Question 2 (Fig. 2). We again varied each parameter by ± 5%, noting if this changed whether polar bear behaviour mitigates or exacerbates ringed seal population growth in years with low ringed-seal productivity. Finally, we also conducted a standard elasticity analysis on the population growth rates in each scenario to assess the impact of changes in individual matrix entries (Caswell and Trevisan 1994; de Kroon et al. 1986).

## Results

Note that all results from our age-structured models are presented by stage for ease of interpretation. For clarity, we only present select results for the comparison scenarios $H_\mathrm{c}$ and $L_\mathrm{c}$ in which we are interested.

### Intermediate results

Several results of secondary importance were obtained throughout our series of calculations, “Hypothetical kill frequencies”, “Stable structure of the population at pre-breeding census”, “Seals available to bears in the spring”, “Number of stage* j* seals eaten” and “Predation mortality, non-predation mortality, and survival”. The right eigenvector ${\mathbf {w}}$ of ${\mathbf {A}}^H$, grouped by stage, implies a stable stage distribution comprised of pups, juveniles, young adults and mature adults in proportions 0.12, 0.47, 0.34,  and 0.07, respectively. The rate of convergence of 10,000 randomly perturbed stage distributions can be seen in Figure S1 (Electronic Supplementary Material). This allows us to assess the appropriateness of our assumption that the population is close to its stable distribution after 10 years.

Assuming a ringed seal population of size $S_{\mathrm{BS}}$ is distributed according to ${\mathbf {w}}$, the total number of female seals immediately following pupping ($\Pr (\text{stage} j)$ at $\tau _2$) in a high-productivity year as calculated from Eq. (3) was $1.19 \times 10^6$, and in a low-productivity year was $1.08 \times 10^6$. The relative availability of pups at this time was twice as high in years of high productivity compared to years with low productivity (Table 2).

The total calories required by polar bears in the Beaufort Sea was estimated from Eq. (13) to be $11.49 \times 10^9$ kcals per year. With our assumption that 2/3 of their calories come from ringed seals ($\theta _{\mathrm{RS}}$ in Table 1), and then that half of that quantity from females, this implies that $3.83 \times 10^9$ calories are obtained by polar bears from female ringed seals. In years of high productivity, ringed seal pups make up the majority of the polar bears’ intake, whether measured in calories or absolute numbers. In years with low productivity, this shifts to the two adult stages (Table 2).

We calculated the predation mortality probability for seals in each stage (Table 2). Combining these estimates of predation mortality and total survival probabilities in high-productivity years, we estimated non-predation mortality (Table 2). From these estimates of predation and non-predation mortality in low-productivity years, we estimated total survival in low-productivity years (Table 2). Compared with high-productivity years, years with low productivity showed increased survival probabilities for pups, and decreased survival for all other stages, most notably for mature adults (Table 2). In contrast, performing the same calculations for the comparison case with constant kill proportions resulted in lower pup survival in years with reduced pup production, and constant survival of the other stages.

### Polar bear stage-specific selection results

**Fig. 3:**
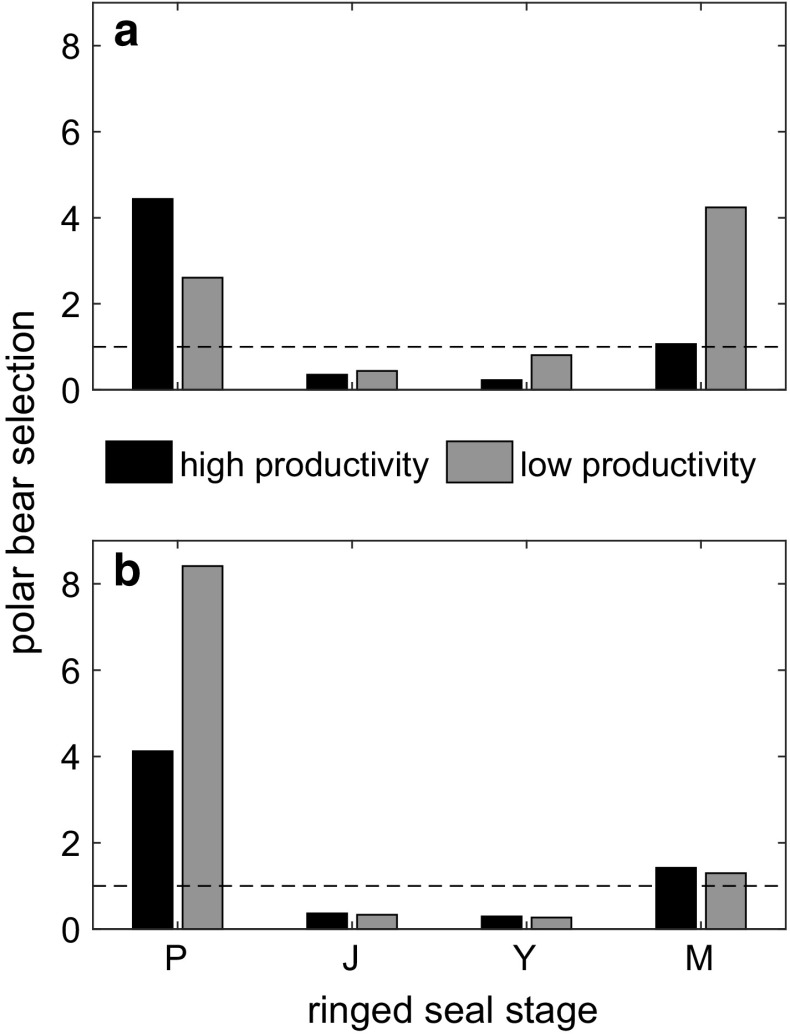
**a** Prey selection (Eq.  9) of polar bears on ringed seal stages (*P* pup, *J* juvenile, *Y* young adult, *M* mature adult), calculated using observations of polar bear kills in years of both high and low ringed-seal productivity. **b** For comparison, selection calculated for a hypothetical scenario in which the composition of polar bear kills is constant for all years. The dotted line at a selection value of 1 signifies neutral preference by bears

To address Question 1 (Fig. 1), we calculated prey selection (Eq. 9) by polar bears on each ringed seal stage in years of both high and low productivity for both observed and comparison kill proportions (Fig. 3). Selection was highest for pups in high-productivity years, and mature adults in low-productivity years. By comparison, if the kill composition was held constant across years, selection for pups doubled in years with low ringed seal productivity.

### Matrix population model results

To address Question 2 (Fig. 1), we analysed matrix population models both with constant environments and with a periodic environment. The growth rate for a constant environment with high ringed-seal productivity was $\log \lambda ^H = 0.021$ (and since ${\mathbf {A}}^H = {\mathbf {A}}^{H_\mathrm{c}}$, $\log \lambda ^{H_\mathrm{c}} = \log \lambda ^H$). In a constant low-productivity environment, $\log \lambda ^L = -\,0.046$, which is slightly higher than the comparison case $\log \lambda ^{L_\mathrm{c}} = -\,0.064$.

The annual growth rate in the periodic environment was $(1/10) \log \lambda ^{B} = 0.0147$, which was slightly lower than that of the comparison case, $(1/10) \log \lambda ^{B_\mathrm{c}} = 0.0151$. The long-term proportions of each stage, according to the periodic model, ranged from 0.07–0.12 for pups, 0.45–0.51 for juveniles, 0.34–0.36 for young adults, and mature adults are between 0.059 and 0.068 (Figure S2, Electronic Supplementary Material).

### Sensitivity analysis results

In no case did varying the parameters in Table 1A by ± 5% alter the pattern of polar bear selection. Varying the parameters in Table 1B by ± 5% altered the population growth rates as expected (e.g. a small increase in the number of bears $B_{\mathrm{BS}}$ resulted in a small decrease in ringed seal population growth). However, some of these small changes did change the order of the periodic growth rates of the observed versus comparison cases, resulting in cases where the annual growth rate in the periodic environment was equal to or slightly higher than the comparison case. From the elasticity analysis, both $\lambda ^H$ and $\lambda ^L$ were most sensitive to changes in juvenile and young adult survival (Table S1, Electronic Supplementary Material). Periodic growth was also most sensitive to changes in juvenile and young adult survival (Table S2, Electronic Supplementary Material).

## Discussion

Theories of prey selection, prey vulnerability, population stability and optimal foraging are common in ecology. Prey switching integrates these concepts, but consistent experimental evidence of the phenomenon is difficult to come by (Murdoch 1969; Sherratt and Harvey 1993). This study suggests a novel type of prey switching—intraspecific prey switching—by comparing changes in the ringed seal stages selected by polar bears in the presence of high and low ringed seal pup availability.

Our main finding was that polar bears selected most heavily for pups and mature seals (as compared with juveniles and young adults) in both high- and low-productivity years. This finding supports the idea that these stages are the most vulnerable to predation. The change in polar bear prey selection from typical years to years with low ringed-seal productivity is suggestive of an intraspecific prey-switching behaviour, where polar bears select for seal pups when they are more abundant, but then display a preference for older ringed seals in years with reduced pup availability. Note that here we use the word preference in its broadest sense, meaning only that the predator consumes proportionately more of one prey type than would be expected given its abundance relative to other available prey, rather than implying a conscious choice made by the predator.

Spatial separation of prey has been proposed as one mechanism leading to prey switching (Hubbard et al. 1982) and may explain the predator preference observed in this study. The switch from selecting for pups to older adult seals in years with low ringed-seal productivity may result from spatial segregation of ringed seals in different stages during the spring (Smith and Stirling 1975; Smith 1987). In years with low ringed-seal productivity, polar bears may leave the fast-ice where ringed seal pups would be found and try their chances with larger, older seals around the ice edge or on the pack ice, where they are more likely to be found. A natural testing of this hypothesis would be to use polar bear telemetry data to look for a shift from land fast-ice to more active ice during spring in years where ringed seals are known to have had low productivity.

In this system, the change in polar bear prey selection reduces the ringed seal population growth rate, though the effect size is small. Compared to the null comparison scenario, this intraspecific switching does, however, result in a larger cohort coming from the year with low productivity by allowing more of the pups to survive by reducing predation pressure on pups. Our null hypothesis was that in years with fewer pups, the pups which are born would experience higher than usual predation. This null hypothesis, explored using the comparative case with constant prey composition, resulted in the expected reduction in annual pup survival $\sigma _{\mathrm{P}}^{L_\mathrm{c}}$ in low productivity years.

Our estimate of population growth in a constant environment with high ringed-seal productivity ($\log \lambda ^H = 0.021$) was slightly lower than two existing estimates for populations with reduced predation pressure, as we would expect. Baltic ringed seals, a population which does not experience predation from polar bears, have an estimated growth rate of 0.045 (Sundqvist et al. 2012). The growth rate of a hypothetical, non-harvested population of ringed seals in the eastern Canadian Arctic was estimated to be 0.0629 (Law 1979).

The periodic comparison model with constant kill proportions predicted slightly higher population growth than the model with switching. This was in spite of the fact that $\lambda ^L > \lambda ^{L_\mathrm{c}}$. In addition to cycles of length 10, we also considered cycles ranging in length from 6 to 12 years, with one low-productivity year per cycle. This result was robust to changes in cycle length; in each case, the comparison model had higher population growth than the model with switching. This non-intuitive result can be explained by considering the stage distribution available to polar bears in the spring. Ringed seal pup numbers are severely reduced in years with low productivity, so even though our results suggest that survival is higher for the pups that are born, this only affects a few individuals, all of which are years away from reproductive maturity. The hypothetical scenario with constant polar bear kill proportions results in reduced pup survival, but the survival of mature adults is higher. These gains in survival probabilities affect individuals who are already contributing to the population through reproduction. While not a large effect, this result highlights the importance of considering environmental sequences as a whole rather than each year in isolation.

Several of our results from the intermediate calculations may be compared to previously published estimates. The annual caloric requirements for the southern Beaufort Sea polar bear population (1800 polar bears) have elsewhere been estimated to be $\approx 4.25 \times 10^9$ kcals (Stirling and Øritsland 1995). Scaled for a population of size $B_{\mathrm{BS}}$, the corresponding estimate is $\approx 7.1 \times 10^9$ kcals per year. Our estimate is approximately 1.6 times that value, which is unsurprising given that our polar bear metabolic rate estimates are $\approx 1.6$ times larger than previous estimates Pagano et al. (2018). In a typical year with high ringed-seal productivity, we estimated that polar bears consume 7% (by number) of the ringed seal population. This is below the range of 14.5–27.5% calculated by Stirling and Øritsland (1995), though they admitted their behavioural method may have overestimated the number of seals consumed by polar bears (Stirling and Øritsland 1995). Our calculation that 29% of ringed seal pups are predated in a typical year falls within the range of 8–44% supplied by Hammill and Smith (1991).

The stable age distribution predicted from matrix ${\mathbf {A}}^H$ had the lowest proportion of seals as pups (12%) and the highest proportion as juveniles (47%), with the remainder falling in the two adult stages (36%). Visual assessment of the convergence of a broad range of perturbed distributions (Figure S1, Electronic Supplementary Material) provided satisfactory evidence that the population would be distributed approximately according to its stable stage distribution 10 years after a perturbation. We would expect the stable age distribution to be reflected in the proportions found during the subsistence open-water harvest, when seals are assumed to be homogeneously distributed and equally susceptible to harvesting (Holst et al. 1999; Smith 1973). Our values are consistent with samples from harvested populations documented by Smith (1987), who reported 15, 54, and 31%, for pups, juveniles, and adults, respectively, as well as Smith (1973), who reported 12, 44, and 43% respectively. Our calculated proportions vary from the harvest proportions reported by Harwood et al. (2012), but those values—21, 14, and 66% for pups, juveniles, and adults—were taken from harvest samples collected earlier in the summer when sampling may have been biased by spatial segregation of seals during breakup when juveniles are thought to be highly mobile, migrating to find high-quality foraging habitat (Freitas et al. 2008). The consistency between our model and observed harvest values provides further justification for assuming the population is distributed approximately according to the stable distribution prior to a year with anomalously late breakup.

We did not consider possible shifts by polar bears to alternative prey species. While species diversity is lower in the eastern Beaufort Sea than in other Arctic regions, polar bears in this region are known to also prey on bearded seals (*Erignathus barbatus*) (Pilfold et al. 2012; Stirling 2002). They may derive a more significant part of their diet from bearded seals to compensate for reductions in the ringed seal population, provided bearded seals do not experience the same years of reduced productivity.

It has also been suggested that in years with low ringed-seal productivity, polar bear populations show signs of stress (reduced numbers, reduced reproductive rates), suggesting that they may consume less energy overall (Stirling and Archibald 1977; Stirling and Øritsland 1995; Stirling and Lunn 1997). Polar bears may also display increased fasting behaviour in response to reduced ringed seal abundance (Cherry et al. 2009; Rode et al. 2018). We also did not consider the effects of fox predation on the ringed seal population. The effects of this may be significant in some years in the Beaufort Sea (Kelly et al. 2010; Smith 1987; Smith et al. 1991), but the timing and causes of surges in fox populations are not well understood.

Being a cryptic species, several of the parameter estimates required for our ringed seal population model were not precisely known. The qualitative nature of the selection results was insensitive to small changes (± 5%) in parameter values, and the response was simply to either reduce or increase predation pressure on ringed seals in an intuitive way. While small parameter changes did result in changes when comparing the periodic population growth rate to that of the comparison model, the magnitude of the difference between these scenarios remained small, emphasizing the point that this behaviour has negligible effect—positive or negative—on the ringed seal population. We also only presented results for one year of reduced ringed seal production per decade. We expect that an extension of our model to include a second or third consecutive year of reduced pup production would yield no new insight and serve only to marginally lower the population growth rate.

One of the reasons that prey switching is difficult to show empirically is that prey switching may occur at one prey density but not at another (Murdoch 1969). We could not explore this possibility here, and similarly could not tease out the effects of relative frequency from absolute frequency. Further, we have only discussed the functional response of the predator (i.e. how the number of prey in each stage eaten changes with prey density) rather than the numerical response of the predator. We have held the predator population size constant across years, which we feel is justifiable when considering short transient periods of reduced ringed-seal productivity.

This study explored this predator–prey system as it was observed over the previous several decades. Since then, the polar bear population in the southern Beaufort Sea has declined (Bromaghin et al. 2015), which we would expect to result in reduced predation pressure on ringed seals. Looking ahead, as the climate warms, the Arctic climatic cycles of the past century are likely to change both in frequency and intensity (Proshutinsky et al. 2015). Environmental fluctuations which affect both predator and prey populations add complexity and nonlinearities to the effects of environmental changes. The response of either prey or predator to both climatic fluctuations and the response of the other could conceivably mitigate or exacerbate anticipated effects of climate change (Wilmers et al. 2007). Over the coming decades, years with low ringed-seal productivity due to heavy winter ice cover and late ice breakup may no longer occur with the same frequency or severity (Kelly et al. 2010). Instead, increased frequency of years with anomalously early breakup may introduce new stresses on ringed seal populations. While this is also believed to have a negative affect on ringed-seal productivity, the mechanism is different, resulting not from low pregnancy rates in females, but from low pup survival rates (Ferguson et al. 2005; Kelly et al. 2010). How the diet composition of polar bears will respond to these changes, remains to be seen.

We have explored how the diet composition of polar bears may have shifted in response to short-term fluctuations in the structure of their prey populations. Spatial segregation of different stages within the ringed seal population provides the most likely explanation for the intraspecific switching type behaviour. While the implications for polar bears, such as associated changes in foraging habitat or increases in hunting effort, may warrant further investigation, the effects on the population growth of their prey appear minor.

### Electronic supplementary material

Below is the link to the electronic supplementary material.

Supplementary material 1 (pdf 119 KB)

## Appendix A: Demographic parameter notes

### $\sigma _{i}^{H}$

Stage-specific survival probabilities are not well known for ringed seals. Considerable variation exists between regions, studies, and calculation methodologies. Smith (1987) calculated annual age-specific survival probabilities from a smoothed age distribution obtained from the summer harvest in the Beaufort Sea. Survival probabilities obtained in this way (with simple averages taken from Table 25 in Smith (1987) to obtain values for each stage) are 0.84, 0.86, and 0.85 for pups, juveniles, and adults, respectively. Survival probabilities obtained from harvest data in this way are notoriously unreliable; however, as they assume that the population was at a stable distribution at the time of sampling (Smith 1987). Other studies have estimated annual pup survival to be markedly lower; 0.61 (Smith 1973), 0.65 (Sundqvist et al. 2012), and 0.69 (McLaren 1958). Estimates of pup survival are complicated by large fluctuations in fox predation pressure (Burns et al. 1982; Lydersen and Gjertz 1986; Smith 1987). Some studies have also suggested increasing mortality for older seals (Smith 1973). The values in our demographic model for typical high-productivity years are informed by the synthesis of studies presented by Kelly et al. (2010) and chosen to be at the upper end of those ranges.

### $m_{i}$

The chosen rates for the expected number of female offsprings produced in a typical year $m_{i}^G$ are comparable to that found elsewhere in the literature, with values increasing from very low at age 4 to approximately 0.4 by age 10 (Hammill 1987; Smith 1973). In the absence of consensus on the topic (see Kelly et al. 2010 for a brief review of the evidence), we do not include sexual senescence in our reproductive rates.

### ${\text{mee}}_{\eta}$

The metabolic energetic equivalent ${\text{mee}}_{\eta}$ of a bear in each stage $\eta$ is a scaling factor based on life-history stage and sex which standardizes the energetic requirements of each bear relative to that of a solitary adult female (Regehr et al. 2015). The eight bear life-history stages (values of $\eta$) are cubs of the year, yearlings, 2 year-old females and males, subadult females and males, and adult females and males. The metabolic energetic equivalents for each stage are 0.2, 0.6, 0.7, 0.9, 0.8, 1, 1, and 1.3, respectively.

### $p_{\eta}$

We use estimates of the age structure of the SB bear population (Table 3 in Stirling and Øritsland 1995) and assume that this structure is appropriate for the entire study region (recall the Beaufort Sea area is comprised of the Northern and Southern Beaufort polar bear subpopulations). We group the number of bears of each age into stages matching those of Regehr et al. (2015). This results in a polar bear population comprised of 10.6% cubs of the year, 9.9% yearlings, 4.6% 2 year-old females, 4.6% 2 year-old males, 8% subadult females, 8.1% subadult males, 27.2% adult females, and 27% adult males.

### FMR

To obtain an average daily FMR value over the year, we used an estimate of 12,324.7 kcal day$^{-1}$ throughout the part of the year where bears are actively hunting on the sea ice, and 8861.2 kcal day$^{-1}$ per day for the approximately 100 days in summer when bears are fasting (Pagano et al. 2018), combining these estimates to obtain an average value of 11375.8 kcal day$^{-1}$ over the whole year.

### *k*_j_^H^, *k*_j_^L^

Observed ringed seal kills for years of typical and low ringed-seal productivity may be seen in Figure 3 of Pilfold et al. (2012). Exact values were obtained through communication with the authors. Note that sample sizes of adult ringed seals killed by bears were low in years with typical ice conditions, and aging to determine whether an adult should be classified as a young or mature adult was not done for all seals. We follow the findings of Pilfold et al. (2012) and assume that the ratio of young adult ($<21$)-to-mature ($\ge 21$) adult seal kills is approximately unity.

Stirling and Øritsland (1995) assumed a constant stage composition of polar bear kills across years, with a similar distribution (61% pups, 22% juveniles, and 17% adults) to that found in high-productivity years by Pilfold et al. (2012).

### cal_j_

We have chosen to use the same values as in Stirling and Øritsland (1995): pups initially provide approximately 10,000 kcals (Apr 1–15), then 50,000 kcals (Apr 16–30), and finally 100,000 kcals (May–Nov). This results in an average estimate of 82,500 kcals for a seal obtained any time in the spring (Apr–July) when most pups are consumed by polar bears.

### S_BS_

A population size of 637,214 ringed seals has been suggested for the Beaufort Sea region (Stirling and Øritsland 1995). However, this value was obtained by simply doubling the number of seals observed hauled out during aerial surveys. This original value was used in an energetics study, where it fit into the authors’ estimates of the number of seals required to sustain the polar bear population. In light of recent work demonstrating that polar bear caloric needs may be $\approx 1.6$ times that previously estimated, we multiply this estimate of the number of seals by 1.6 as well, resulting in our estimate of $\approx$ one million ringed seals. We assume the overall sex ratio of ringed seals to be 1:1 (McLaren 1958; Smith 1973), so we use an estimate of 500,000 female ringed seals.

## Appendix B: Derivation of ($\# \text{ stage }{} { j} \text{ seals } \text{eaten}$) in Eq. (5)

In the following, note that parameters with estimates in the literature are denoted with braces, and descriptions of their values and sources are in Table 1. To obtain an estimate of the number of seals in each stage that are eaten, we worked in terms of calories rather than numbers of ringed seals (RSs) consumed by polar bears (PBs) in the Beaufort Sea (BS). This was necessary because not all ringed seals are of equal caloric value to a polar bear. We let

12 $$\begin{aligned} & \# \text{ stage } {} { j} \text{ seals } \text{eaten} \nonumber \\&\quad = (\!\text{calories } \text{ from } \text{ RSs } \text{ required } \text{ by } \text{ PB } \text{ population\!}) \nonumber \\&\quad\quad \times (\!\text{ proportion } \text{ of } \text{ calories } \text{ from } \text{stage } {} { j} \text{ RSs }\!)/ \nonumber \\&\quad\quad (\underbrace{{\mathrm{calories\, gained\, per\, stage\, }}{} { j} \text{ RS } }_{{\text{cal}}_j}\!). \end{aligned}$$

Here

13 $$\begin{aligned}&\text{ calories } \text{ from } \text{ RSs } \text{ required } \text{ by } \text{ PB } \text{ population } \nonumber \\&\quad = \left( \sum _\eta {\text{ calories } \text{ required } \text{ by } \text{ all } \text{stage } \eta \text{ PBs }}\right) \nonumber \\&\quad\quad \times \frac{1}{2}(\underbrace{{\text {proportion of calories PBs obtain from RSs}}}_{\theta _{\mathrm{RS}}}), \end{aligned}$$

where $\eta$ ranges through the 8 distinct polar bear stage and sex classifications (cubs of the year, yearlings, 2 year-old males and females, subadult males and females, and adult males and females) as described in Regehr et al. (2015). The factor of one half is included to account for our female-only model; we assumed polar bears kill with an approximately 1:1 sex ratio (Pilfold et al. 2012), so approximately half of their caloric needs come from female seals. To estimate caloric demands, we used polar bear field metabolic rates (FMR). We assumed

$$\begin{aligned}&\text{ calories } \text{ required } \text{ by } \text{ all } \text{stage } \eta \text{ PBs } \nonumber \\&\quad = (\# \text{ of } \text{stage } \eta \text{ PBs } \text{ in } \text{ BS \!})(\!\text{ FMR }\!)(365 \text{ days \!})\nonumber \\&\quad \times (\underbrace{{\text {metabolic energetic equivalence of stage }}\eta \text{ PBs }}_{{\text{mee}}_{\eta} }), \end{aligned}$$

where the metabolic energetic equivalence is a scaling factor based on life-history stage and sex which standardizes the energetic requirements of each bear relative to that of a solitary adult female (Regehr et al. 2015). The

$$\begin{aligned} \# \, \text{stage}\, \eta \text{ PBs } \text{ in } \text{ BS } = (\underbrace{\%\,{\mathrm{stage }}\, \eta \text{ PBs } \text{ in } \text{ BS }}_{p_{\eta} })(\underbrace{\#\,{\mathrm{PBs\, in\, BS}}}_{B_{\mathrm{BS}}}). \end{aligned}$$

To relate (proportion of calories from stage *j* RSs) in Eq. (12) to the relative kill frequencies found by Pilfold et al. (2012), we assumed

14 $$\begin{aligned}&\text{ proportion of calories from stage } { j} \text{ RSs} \\ &= \frac{\text{observed calories from stage } { j} \text{ RSs in kills total observed calories in kills }} \nonumber \\ &= \frac{(\text{observed }\#\text{ stage } {j} \text{ kills\! })(\!\text{ calories gained per stage } { j} \text{ RS})}{\sum _\ell {\left[ (\text{observed }\#\text{stage } \ell \text{ kills \!})(\!\text{ calories gained per stage } \ell \text{ RS})\right] }} \end{aligned}$$

where $\ell$ runs through each ringed-seal stage. Substituting this into Eq. (12) and simplifying yielded

$$\begin{aligned} \begin{aligned} \# \text{ stage }{} { j} \text{ seals eaten }&= \frac{(\!\text{ calories from RSs required by PB population })(\overbrace{\text{ observed } \# \text{ stage } { j} \text{ kills}}^{k_j}\!)}{\sum _\ell {\left[ (\underbrace{{\mathrm{observed}}\,\# \text{ stage} \,\ell \text{ kills }}_{k_j})(\underbrace{{\mathrm{calories\, gained\, per\, stage\, }}\ell \text{ RS }}_{{\text{cal}}_\ell })\right] }} \end{aligned} \end{aligned}$$

where $\ell$ again runs through each stage. All of this together, simplified, resulted in Eq. (5).
