# Supplementary material for: Evidence of intraspecific prey switching: stage-structured predation of polar bears on ringed seals
Source: Oecologia. 2018 Nov 19;189(1):133–48. doi: 10.1007/s00442-018-4297-x (PMC6323094; doi:10.1007/s00442-018-4297-x)
Supplement: Supplementary file 1 — Supplementary material 1 (pdf 119 KB) [file 442_2018_4297_MOESM1_ESM.pdf]

Supplementary material to “Evidence of intraspecific prey switching: stage-structured predation of polar bears on ringed seals”

Jody R. Reimer†, Hannah Brown, Elaine Beltaos-Kerr, Gerda de Vries

† Corresponding author. [email] jrreimer@ualberta.ca. [phone] +1.780.492.3308. [fax] +1.780.492.9234

Table S1: Elasticity of the growth rate for $\mathbf{A}^H$ and $\mathbf{A}^L$ to reproductive rates and survival, grouped by stage.

elasticity of	to parameter	value by stage j			
		P	J	YA	MA
λ^H	m_j^H	-	0.01	0.05	0.01
	σ_j^H	0.07	0.40	0.40	0.06
λ^L	m_j^L	-	0.01	0.05	0.01
	σ_j^L	0.07	0.40	0.42	0.03

Table S2: Elasticity of the asymptotic growth rate of the decadal periodic matrix model Eq.(11) to changes in each of the component matrices, grouped by stage. Note that for each of the 9 years of high productivity, the elasticity of λ^B to m_j^H was the same at the recorded precision. Notation is consistent with $\mathbf{B} = \mathbf{A}^L \underbrace{\mathbf{A}^H}_{\#9} \dots \underbrace{\mathbf{A}^H}_{\#1}$.

elasticity of	to parameter	value by stage j			
		P	J	YA	MA
λ^B	m_j^H (# 1-9)	-	0.01	0.05	0.01
	m_j^L	-	0.01	0.03	0.01
	σ_j^H (# 1)	0.04	0.43	0.40	0.06
	σ_j^H (# 2)	0.07	0.39	0.41	0.05
	σ_j^H (# 3)	0.07	0.39	0.41	0.05
	σ_j^H (# 4)	0.07	0.38	0.41	0.06
	σ_j^H (# 5)	0.07	0.38	0.41	0.06
	σ_j^H (# 6)	0.07	0.39	0.41	0.06
	σ_j^H (# 7)	0.08	0.39	0.40	0.06
	σ_j^H (# 8)	0.07	0.42	0.38	0.06
	σ_j^H (# 9)	0.07	0.42	0.38	0.05
	σ_j^L	0.07	0.43	0.40	0.06

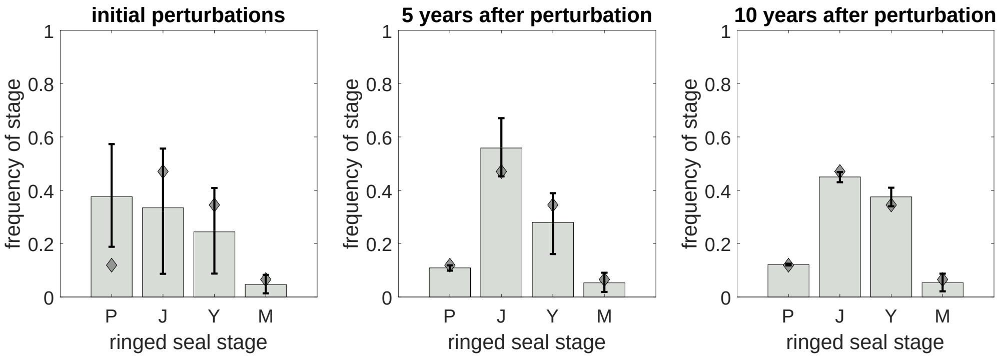

Fig. S1 Convergence of 10000 randomly perturbed stage distributions towards a stable distribution (see text for details of the perturbation). Bars show the median frequency for each of four ringed seal stages (pups, juveniles, young adults, and mature adults) and black bars represent the middle 95th percentile of the 10000 simulations. The dark grey diamonds (the same in all three plots) are the stable stage distribution of the population under the constant, high fertility environment ($\mathbf{A}^H$)

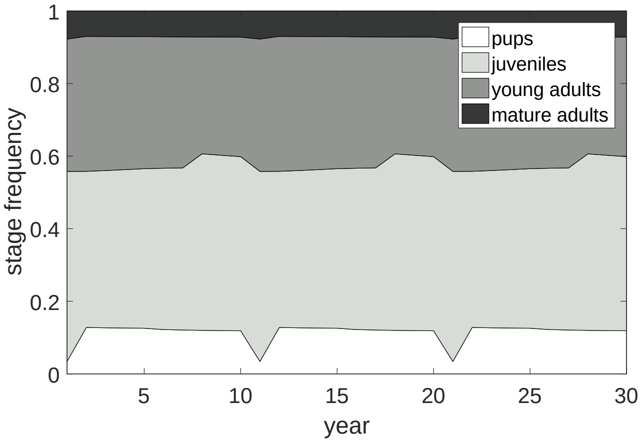

Fig. S2 Stable distribution of the age-structured matrix model (Eq.(11)) for ringed seals, with ages classified into four distinct stages. The population experiences a periodic environment over 10 years, with 9 high productivity years and 1 low productivity year. In low productivity years, ringed seals also experience corresponding changes in survival due to predation pressure from polar bears.
